# Analysis of Land Use Change and Urbanization in the Kucukcekmece Water Basin (Istanbul, Turkey) with Temporal Satellite Data using Remote Sensing and GIS

**DOI:** 10.3390/s8117213

**Published:** 2008-11-13

**Authors:** H. Gonca Coskun, Ugur Alganci, Gokce Usta

**Affiliations:** Istanbul Technical University, Faculty of Civil Engineering, Department of Remote Sensing 34469 Maslak, Istanbul, Turkey; E-mails: alganci@itu.edu.tr; gokceusta@gmail.com

**Keywords:** Remote sensing, GIS, water basin, urban monitoring, image analysis and land use classification

## Abstract

Accurate and timely information about land use and land cover (LULC) and its changes in urban areas are crucial for urban land management decision-making, ecosystem monitoring and urban planning. Also, monitoring and representation of urban sprawl and its effects on the LULC patterns and hydrological processes of an urbanized watershed is an essential part of water resource planning and management. This paper presents an image analysis study using multi temporal digital satellite imagery of LULC and changes in the Kucukcekmece Watershed (Metropolitan Istanbul, Turkey) from 1992 to 2006. The Kucukcekmece Basin includes portions of the Kucukcekmece District within the municipality of Istanbul so it faces a dramatic urbanization. An urban monitoring analysis approach was first used to implement a land cover classification. A change detection method controlled with ground truth information was then used to determine changes in land cover. During the study period, the variability and magnitude of hydrological components based on land-use patterns were cumulatively influenced by urban sprawl in the watershed. The proposed approach, which uses a combination of Remote Sensing (RS) and Geographical Information System (GIS) techniques, is an effective tool that enhances land-use monitoring, planning, and management of urbanized watersheds.

## 1. Introduction

Istanbul and the Bosphorus are among most valuable places in the world with their natural beauty and important historical heritage. Three sides of Istanbul are surrounded by sea. The population of the Istanbul metropolitan area has increased rapidly because of continuous immigration from other regions, and according to the Turkish Statistical Institute data for the year 2000, Istanbul's population had exceeded 10 million [13]. Istanbul faces some significant administrative, social, economic and drinking water pollution problems [16]. Periodical repeated urban monitoring is necessary for the protection of the drinking water dam catchments [9].

The closeness of the water reservoirs to the city and district center brings down the cost of supplying water to the users, but entails difficulties in the protection and management of the water resource basins against illegal and unplanned urbanization [8]. The process of urbanization or its growth drives changes in land use/cover patterns, which also have adverse impacts on the ecology of the area, especially hydro-geomorphology and vegetation [10]. The city's settlement and industrial wastes are partly treated; most of the polluted water is discharged into the Bosphorus but some goes into rivers which then feed drinking water dams, as occurs in other developed and developing countries. In recent years, the problem has been mostly eliminated with the construction of a large number of treatment facilities built the Istanbul Water and Sewerage Administration (ISKI), but pollution loads with negative effects on Istanbul's water still come from settlements and industries as point and non-point sources, bilge water discharged from ships, and also polluted rivers and total suspended sediment due to erosion.

Today, satellite data enables periodically repeated analysis and identification of the urban changes in the city and catchments areas and pollution of sea (water) more accurately and rapidly [2, 3]. In short, there have been significant advances in the development of space-based tools that offer rapid, repeated, and concurrent synoptic assessment of environmental parameters in oceanic and adjacent land areas [12, 15, 17]. Negative impacts of rapid urbanization on water basins can be analyzed using multitemporal satellite imagery with change detection techniques. The pollution of water has a negative effect on human health, plants and water life. Monitoring of environmental changes, exposed pollutants and their reasons and finding needed precautions should be a must. RS is an important alternative for such monitoring of changes in urban, water and air [4-6, 11, 14].

The main objective of this paper is to describe the application of multi-temporal satellite data for monitoring the land-use and land-cover (LULC) distribution of the Kucukcekmece Water Basin using RS and GIS techniques. The ultimate aim is to present the utility of such modern techniques of RS and GIS in determining the LULC changes within time especially when large watersheds are concerned. This investigation presents temporal urbanization analysis of Kucukcekmece Water Basin using the results of merging and image processing of different spectral and spatial resolution data.

RS data have been transformed into the UTM coordinate system. Image enhancement, merge and classification techniques have been applied to the images and raster- vector integration was done for GIS queries. Time dependent changes of settlements at water basins areas were analyzed by taking urbanization in the absolute, short, medium and long protection zones as a base. LULC change results for Kucukcekmece Catchment are shown both in graphical and numerical form. Three-dimensional models of the water basin were formed and considered together with the resultant images processed by different image processing methods.

## 2. Study Area

**Figure 1.**
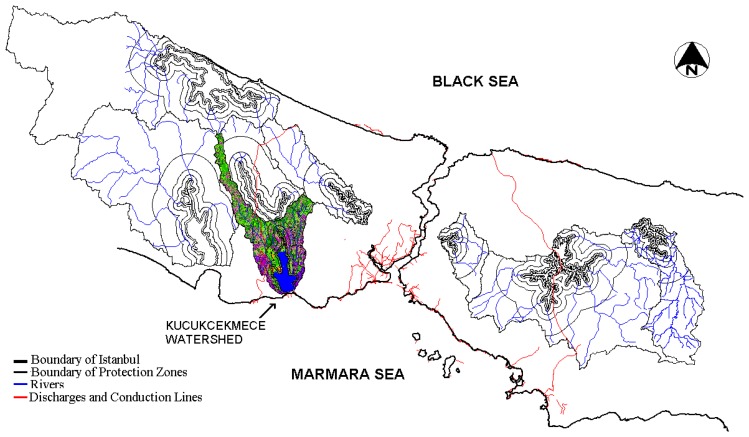
Location of Kucukcekmece Water Basin.

The geographical coordinates of the project area that covers the Bosphorus and the water basins are latitude 40° 60′ north and longitude 41° 70′ west. The location of the Kucukcekmece Water Basin within the borders of Metropolitan Istanbul is shown in Figure 1. The total Kucukcekmece catchment area is about 340 km^2^. Kucukcekmece Lake Basin is 15 km away from the west of the city centre and located between the western border of Kucukcekmece District and the eastern border of Avcilar District. The morphologic structure of Kucukcekmece Lake is that of a natural lagoon. The area of Lake Basin is 16 km^2^ and its maximum depth is 20 m at the south part of the lake. Hadimkoy, Sazli and Nakkas streams feed the lake. The town of Kucukcekmece has been experiencing rapid urbanization, with a high rate of migration during the last two decades. Much of the urbanization around the lake has been unplanned. The population of the town in the 1930s was 707, and since then it has increased considerably, reaching 350,000 in the 1990s and about 600,000 in 2000. Consequently, the lagoon has been subjected to heavy nutrient inputs because of the poor sanitary treatment of wastewater associated with human population growth around the lake. In addition, a considerable amount of industrial waste has been introduced directly into the lagoon.

## 3. Methodology

### 3.1 Image Processing

During the analyses of urban changes in Kucukcekmece Water Basin, Landsat-5 TM, SPOT-XS and Pan, IRS-1C and IRS-LISS, Landsat-5 TM satellite data were used, respectively, for the years of 1992, 1993, 2000 and 2006. The spatial resolutions for SPOT-PAN, XS are 10 m and 20 m, for IRS1-C/D, IRS LISS they are 5.8 m and 23.5 m, and 30 m for Landsat TM.

In this study, 1:25,000, 1:5,000 scaled digital maps that cover the study area and orthophotos formed of 1:5,000 air photos were used as ground truth. Digital maps were used for rectification of the satellite imageries.

**Figure 2.**
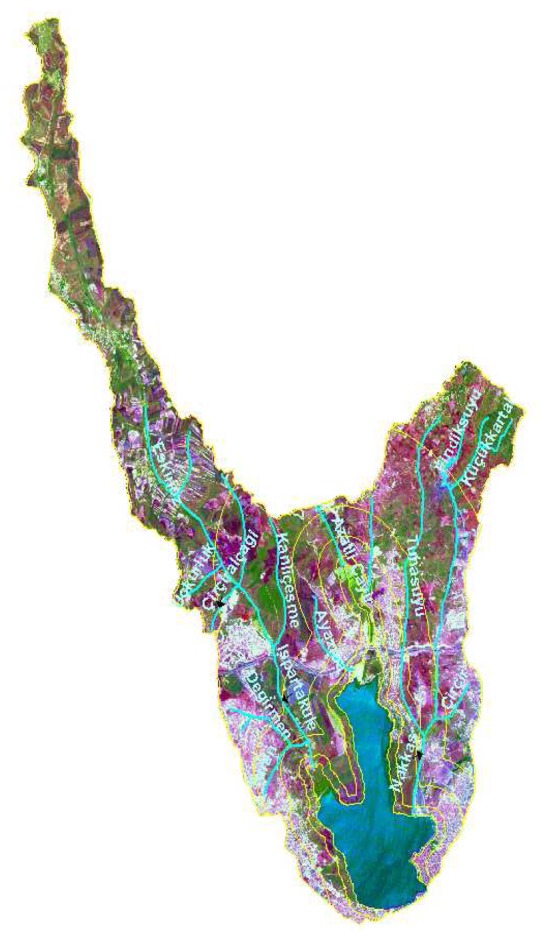
Result of merge operation using Landsat TM and SPOT- PAN Satellite data, for Kucukcekmece Water Basin dated 1992 with fitted streams and protection zones on as vector data.

First, the digital satellite data set was transformed into the UTM International 1909 Zone 35 coordinate system using the 1:5,000 digital topographic maps in order to achieve the necessary geometric registration. Taking 50 ground control points from the maps, the images were geometrically corrected before applying image merging and classification. From a test of the registration accuracy on the test points the resulting root mean error (RMSE) amounted to ±0.5 pixels. A coordinate transformation was applied using a polynomial transformation equation and resampling was done using the cubic convolution algorithm. Appropriate image enhancement techniques were applied for all rectified satellite images band by band. The IHS transformation method was used to obtain merged, multi-sensor images. High-resolution PAN digital satellite data was combined with multi-spectral data [2]. This transformation method displays advantageously results including quantitative and qualitative digital image analysis (Figure 2).

### 3.2 Land Use Classification

There are two primary methods of image classification utilized by image analysts, which are unsupervised and supervised classification. Unsupervised image classification is a method in which the image interpreting software separates the pixels in an image based upon their reflectance values into classes or clusters with no direction from the analyst. Supervised image classification is a method in which the analyst defines small areas, called training sites, on the image which are representative of each desired land cover category. The delineation of training areas representatives of a cover type is most effective when an image analyst has knowledge of the geography of a region and experience with the spectral properties of the cover classes. The image analyst then trains the software to recognize spectral values or signatures associated with the training sites. After the signatures for each land cover category have been defined, the software then uses these signatures to classify the remaining pixels [8].

Various classification methods were applied to the rectified and enhanced multispectral data to analyze different terrain and structure characteristics. First, ISODATA, one of the unsupervised classification methods, was carried out individually for all images with 0.95 threshold value and maximum iteration number 20, and then 50 clusters were obtained. Because of the similarity of reflected values between excavation sites, uncultivated lands, urban areas and roads, the efficiency of the unsupervised classification was very poor. Therefore a supervised classification method was applied. Orthophotos that contains the near time structuring in water basins were used for classification as ground truth. In addition, field works were done in the study areas. There had been some difficulties about past residential data because 1992, 1993 years were available to be analyzed in the project. These difficulties were eliminated, especially in residential areas, by using 1996 dated orthophotos and getting samples of the known structures from the preceding classified images.

**Figure 3.**
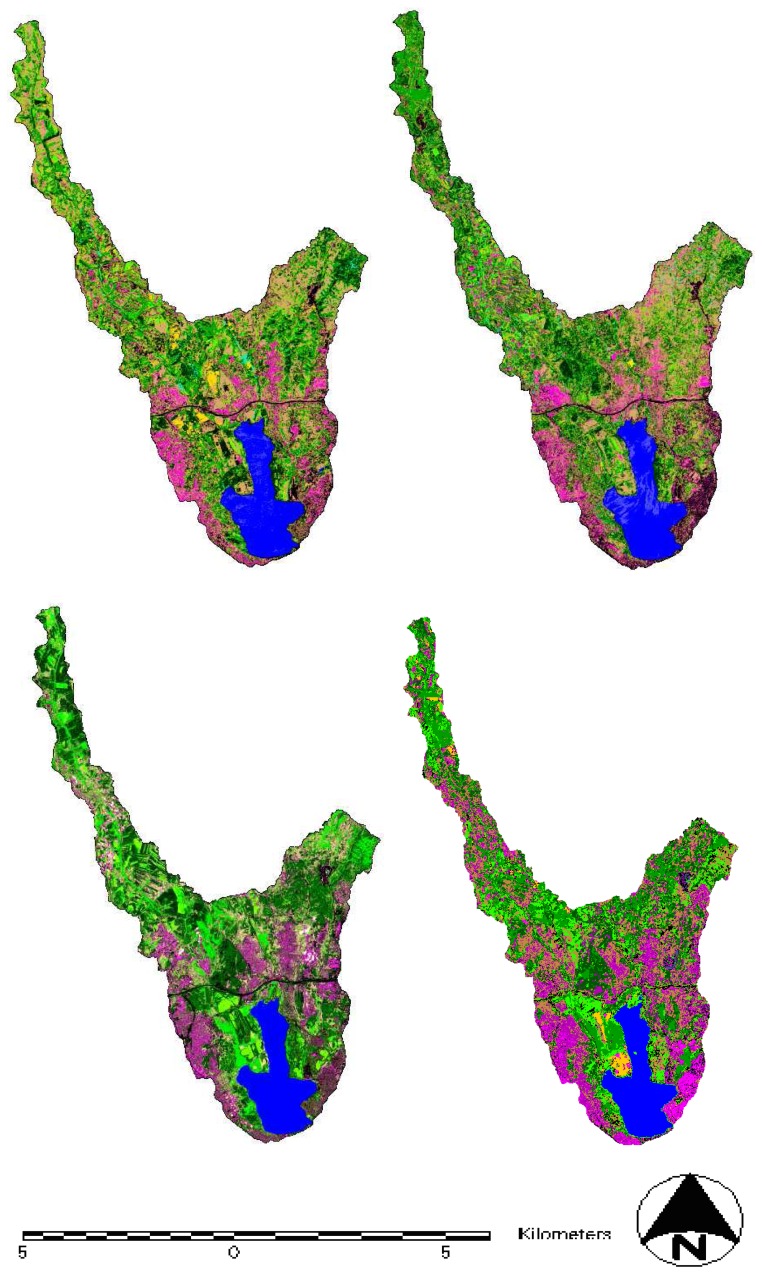
Classified images of the Kucukcekmece Water Basin dated 1992, 1993, 2000 and 2006.

**Figure 4.**
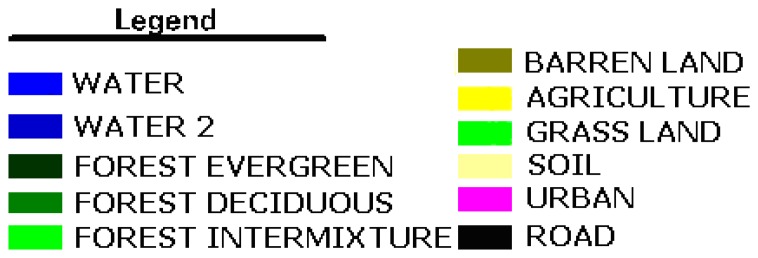
Legend for Land Cover / Land Use classification.

For this supervised classification, the Maximum Likelihood (Bayesian) decision rule was used. New samples from the mixed areas were selected according to the ground truth data and added to the signature file that was formed by the unsupervised classification and the cluster number was increased to 80. In the next step, these 80 clusters were reduced to eleven final classes with recode operation. These eleven classes were verified by confirmation through the ground samples taken from the experimental area with accurate field information for near time. Resultant classified images are shown in Figure 3 for the studied years. The legend for the classes is shown in Figure 4. The classification results were evaluated in an accuracy assessment. In this accuracy assessment, for each classification up to four different years, 100 random pixels were chosen and these pixels were compared with the results of the fieldwork. In this project for Kucukcekmece, the user accuracy was found as 84%, 84%, 83.33%, and 86% respectively for the years 1992, 1993, 2000 and 2006 satellite data with randomly selected 100 points.

### 3.3 GIS Modeling Using Raster-Vector Integration

Geographical Information System (GIS) was used in this research in order to combine raster, vector and ground truth data. Using the GIS capabilities of overlaying these different types of data and with the capability of query function, LULC analyses were done for selected years. Temporal urban changes were traced visually and statistical analysis between studied years was done with the model structured with GIS. A vector-raster data integration was done on the Kucukcekmece Basin. In the modeling, 9 vector data layers and 247 raster data layers were constituted for GIS. The recorded information and geocorrected images of water basins and dams, protection zones and area calculations were transferred to these layers.

One part or the whole of the demand area can be seen with the image base and any vector data can be opened on these bases. So, the analyses can be done by combination of base images and desired vector data. Users can examine the water basin entirely or select the protection zone as merged or classified image, and also can reach settlement area information in hectare unit according to the years 1992, 1993, 2000 and 2006.

**Figure 5.**
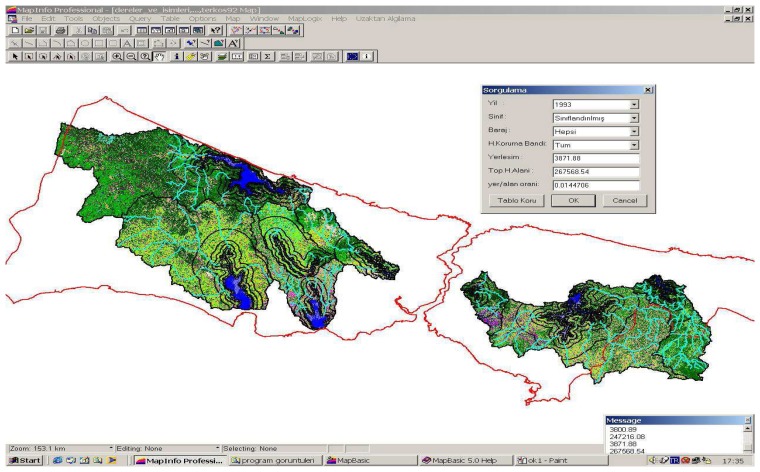
A GIS query that shows the analysis of time dependent changes of urbanization in hectares and its ratio to total basin area [1].

The project entitled “Monitoring of Istanbul Drinking Water Dam's Water Basins with Multitemporal Satellite Data” and carried out by Istanbul Technical University for ISKI, was been considered in this paper. Under the project agreement, time dependent changes of settlements at water basins areas were analyzed by taking urbanization in the absolute, short, medium and long protection zones as a base. In this paper only the Kucukcekmece Basin was taken as an example. However, all of the water basins are shown in the GIS query in Figure 5.

**Figure 6.**
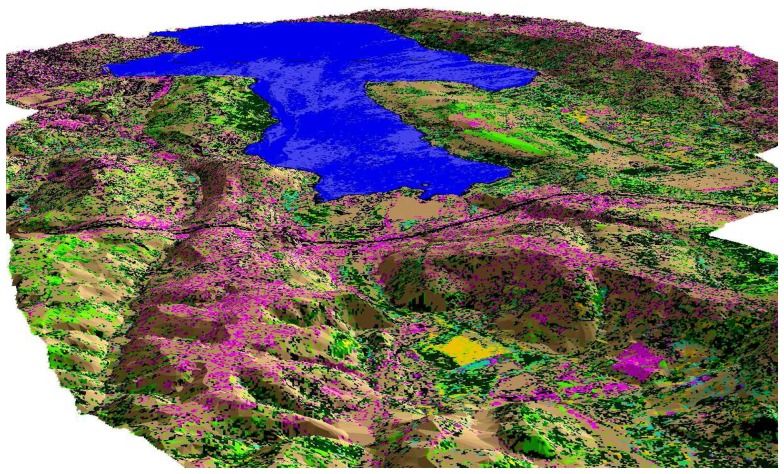
A north side looking 3-D model of Kucukcekmece Water Basin constructed with the drape of 1993 dated classified image over DEM.

In order to create the digital elevation model (DEM), a Triangulated Irregular Network (TIN) was constructed from contour map. This TIN model was then used to extract x, y, and z information of the basin. The height map was formed with the contour interval and color scale and the slope map. The DEM of the basin was then created and classified image was draped over this DEM to form a 3D model presented in Figure 6. This 3D model was used for visual interpretation of the topographic structure of Kucukcekmece Water Basin which makes it possible for the analyst to determine the hydrologic characteristics of the basin and rivers.

## 4. Results and Discussion

Monitoring of environmental protection areas of the water basins by urban planners implies the collection of updated systematic information on LULC patterns and infrastructure facilities. Periodically repeated total field inventories are necessary to protect the drinking water dam catchments areas against heavy pollution. RS and GIS techniques are important tools for determining and updating LULC changes at water basin areas. GIS provide not only a common environment for spatial data but also form a powerful technology for the quantitative and qualitative analysis of the LULC for the map updating.

In the year 2006, the protection zones around the drinking water resources were determined by ISKI. ISKI indicates activities that are permissible in each zone: the absolute, short, medium and long-range protection zones [7]. RS and GIS methods are applied for illustration in established models on the protected four zones of the Kucukcekmece Water Basin.

This study is aimed to monitor urban changes with remote sensing data. Geometrically corrected and enhanced satellite dataset are subjected to a classification process. After the classification, obtained LULC classes are controlled with accuracy assessment operation. With randomly selected 100 random pixels compared with field work, the user accuracy was found to be 84%, 84%, 83.33% and 86%, respectively, for the 1992, 1993, 2000 and 2006 satellite data.

It was shown that LULC changes in the Kucukcekmece Water Basin for the years 1992, 1993, 2000 and 2006 can be identified with this methodology. As seen in Table 1, there was an increase in urban areas between these years.

The uncontrolled development of urbanization, especially on the south part of the basin and motorway construction at the north part of the basin has caused shoreline changes. The urban area increased by 341.34 ha between 1992 and 1993. This difference is equivalent to a 46.83% increase in one year. The urban area decreased by 80.98 ha between 1993 and 2000 due to the evacuation of illegal housing by ISKI. This evacuation amount corresponds to 7.6% of the total urban area. However, the urban increase of 2,637.96 ha between 2000-2006 dates give a dramatic augmentation with 266.72 % on total urban area in six years. Despite the negative effects of 1999 earthquake, there was an extreme increase in urban areas in this 14 year period. This land use increase extends both to urban and rural areas.

This study shows that, remotely sensed data combined with ground truth data makes it possible to explore the LULC management problems associated with the future rapid growth of the Kucukcekmece population. A joint application of Remote Sensing with GIS provided assembling the study and query.

Usually, the major land-use change is caused by the increasing demand for non-agricultural land because of urban and manufacturing development. Urban sprawl is increasingly considered a significant and growing problem that entails a wide range of social and environmental costs [10]. Uncontrolled population growth and the rapid development of world-wide urbanization have an extreme impact on the environment. It is obvious that, rapid, uncontrolled and illegal urbanization brings out domestic and industrial pollution loads via streams due to insufficient treatment facilities. To minimize effectively the negative impacts to the environment, and make a control mechanism for population growth, there is a need for accurate, reliable and up to date data at regular intervals. Remote Sensing technology can serve as a basic data source for this need.

Land use plans should be prepared in accordance with a protection strategy. Local governments, relevant administrations, municipalities, planning and environmental protection agencies must protect the catchments area for protection of this precious reserve of good drinking water. They must cooperate with universities and scientific organizations; work in harmony under good co-ordination, rather than attempting isolated solutions on an individual basis. Adaptation of the protection strategy to the current status requires certain, urgent and short-term measures. Future generations should be considered, and land use activities, which can result in irreversible changes, should be kept under control.

## Figures and Tables

**Table 1.** The urbanization analysis of Kucukcekmece Water Basin. Units are in hectares.

Year	Urban (U)	Total Basin Area (TBA)	% (U/TBA)

**1992**	728.85	16,308.77	4.47
**1993**	1,070.19	16,308.77	6.56
**2000**	989.021	16,308.77	6.06
**2006**	3,626.980	16,308.77	22.24
